# Trainers' Attitudes towards Cardiopulmonary Resuscitation, Current Care Guidelines, and Training

**DOI:** 10.1155/2016/3701468

**Published:** 2016-04-06

**Authors:** M. Mäkinen, M. Castrén, J. Nurmi, L. Niemi-Murola

**Affiliations:** ^1^Department of Anaesthesia and Intensive Care Medicine, Helsinki University Hospital, HUS, Stenbäckinkatu 9, 00029 Helsinki, Finland; ^2^Department of Clinical Science and Education and Section of Emergency Medicine, Karolinska Institutet, Södersjukhuset, Solnavägen 1, 17177 Stockholm, Sweden; ^3^Department of Emergency Medicine, Helsinki University Hospital, HUS, Stenbäckinkatu 9, 00029 Helsinki, Finland; ^4^Faculty of Medicine, University of Helsinki, Haartmaninkatu 8, 6300014 Helsinki, Finland

## Abstract

*Objectives*. Studies have shown that healthcare personnel hesitate to perform defibrillation due to individual or organisational attitudes. We aimed to assess trainers' attitudes towards cardiopulmonary resuscitation and defibrillation (CPR-D), Current Care Guidelines, and associated training.* Methods*. A questionnaire was distributed to CPR trainers attending seminars in Finland (*N* = 185) focusing on the updated national Current Care Guidelines 2011. The questions were answered using Likert scale (1 = totally disagree, 7 = totally agree). Factor loading of the questionnaire was made using maximum likelihood analysis and varimax rotation. Seven scales were constructed (*Hesitation*,* Nurse's Role*,* Nontechnical Skill*,* Usefulness*,* Restrictions*,* Personal*, and* Organisation*). Cronbach's alphas were 0.92–0.51. Statistics were Student's *t*-test, ANOVA, stepwise regression analysis, and Pearson Correlation.* Results*. The questionnaire was returned by 124/185, 67% CPR trainers, of whom two-thirds felt that their undergraduate training in CPR-D had not been adequate. Satisfaction with undergraduate defibrillation training correlated with the* Nontechnical Skills* scale (*p* < 0.01). Participants scoring high on* Hesitation* scale (*p* < 0.01) were less confident about their* Nurse's Role* (*p* < 0.01) and* Nontechnical Skills* (*p* < 0.01).* Conclusion*. Quality of undergraduate education affects the work of CPR trainers and some feel uncertain of defibrillation. The train-the-trainers courses and undergraduate medical education should focus more on practical scenarios with defibrillators and nontechnical skills.

## 1. Introduction

Finnish national Current Care Guidelines for cardiopulmonary resuscitation based on ERC guidelines were first published in 2002 and updated in 2005, 2011, and 2015 [1]. The goal of these guidelines was to enable nurses as well as physicians to use an automated external defibrillator (AED). Although the published guidelines introduce newly developed resuscitation techniques to clinical practice, the outcome of cardiac arrest patients has not significantly improved [1]. The implementation of these guidelines has been studied extensively in Finland [2–7]. Special focus has been nurses' and students' ability to implement the guideline recommendations in clinical practice. Several studies have revealed deficiencies in cardiopulmonary resuscitation and defibrillation (CPR-D) skills of nursing students [5, 8], nurses [6, 8–10], and also physicians [11, 12] and anaesthesiologists [13]. Effective education programmes are needed for the wide implementation of rapid defibrillation and the use of AEDs [14, 15].

The basic education of nurses has poorly prepared them for the CPR and leadership [4, 6]. This increases the demand for changes in training, regular training, and evaluation in the workplace. In spite of a structured training program and availability of equipment at their workplace, nurses hesitate to start CPR and do not necessarily use defibrillators in resuscitation situations because of lack of confidence, the fear of harming the patient, and perceived difficulty in interpreting electrocardiograph rhythms [4, 5, 14, 16]. This perceived reluctance to perform defibrillation may be due to individual or organisational attitudes [5–7, 14], which have been shown to influence professional behavior [4, 6, 7, 14].

According to our previous study, implementation of a training program increased perceived negative organisational attitudes towards nurse-performed defibrillation and the nurses felt unsure of their role in CPR-D situation [4]. This indicates a problem in the healthcare organisation itself, the delivery of educational programme [1, 11], or the unwritten and unofficial curriculum, for example, hidden curriculum [17].

*Aim of the Study*. The aim of this study was to evaluate trainers' attitudes towards cardiopulmonary resuscitation and defibrillation (CPR-D), Current Care Guidelines, and training using a structured questionnaire answered by the trainees [4].

## 2. Methods

### 2.1. Sample and Data Collection

In spring 2011 a questionnaire was distributed to all participants of local CPR trainers' education seminars in Finland. The seminars were aimed for CPR trainers in southern Finland: Sample 1, *n* = 105, Jyväskylä; Sample 2, *n* = 80, Seinäjoki, (*N* = 185), focusing on the updated guidelines for CPR published a month earlier 2011. The anonymous answers were collected during the seminars and some were later returned by mail. The questionnaire has been used in previous studies [4, 7]. Attitudes towards CPR-D and attitudes towards Current Care Guidelines were analysed separately.

### 2.2. Ethics

The respondents were informed about the interests of the study and the data collection. The participation in the study was completely voluntary. The ethical principles for medical research which involved human subjects were followed [18].

### 2.3. Measures

The questionnaire was constructed to investigate attitudes towards CPR-D, Current Care Guidelines, and training. In the questionnaire, there were six questions about demographic and nine about educational background, 33 attitudes-related items, which included a validated Attitude towards Guidelines Scale (AGS) [19], and 10 questions based on national 2011 CPG guidelines for CPR training, totalling 58 items. A 7-point Likert scale was used to measure attitudes. Respondents rated their agreement with each item on a 7-point Likert scale ranging from strongly totally disagree to strongly agree, 1 = totally disagree, 2 = somewhat disagree, 3 = slightly disagree 4 = neither agree nor disagree, 5 = slightly agree, 6 = somewhat agree, and 7 = totally agree [20].

### 2.4. Data Analysis

The statistical analyses were carried out by the SPSS 17.0 (SPSS 17.0 SPSS Inc., Chicago, IL, USA) for windows version software package. Factor loading of the questionnaire was made using maximum likelihood analysis and varimax rotation to examine the underlying constructs of the survey instrument. The items concerning attitudes and the AGS items [19] were analysed separately. Three scales were constructed (*Hesitation*,* Nurse's Role*, and* Nontechnical Skill*) (Table 1) about attitudes towards CPR and four about the AGS items (*Usefulness*,* Restrictions*,* Personal*, and* Organisation*) (Table 2). Reliability of the questionnaire was calculated using Cronbach's alphas, which were 0.92–0.51. Statistics: the data were analysed by means and tested by parametric and nonparametric tests, Student's *t*-test, ANOVA, Pearson Correlation, and Regression analysis.

## 3. Results

The final sample consisted of 124/185 (67%) participants who anonymously answered the questionnaire sampled in two batches. Sample 1 (61/105) were mainly trainers working in primary care. The majority of Sample 2 (63/80) trainers were working in secondary hospitals and some also in tertiary hospitals, representing wards of internal medicine, surgery, paediatrics, geriatrics, and gynaecology/obstetrics and emergency room. The mean age of respondents was 44 years. Their background data is presented in Table 3. Seventy-eight percent (94/124) were nurses. One-third of the respondents graded their undergraduate education in CPR-D as adequate. Of the trainers 63.7% (79/124) were dissatisfied about their undergraduate CPR training and 70.1% (87/124) about defibrillation training and 78% (97/124) thought that education about rhythms was not adequate. Ten percent had participated in CPR-D education during their spare time and at their own expense.

Educational background affected respondents' attitudes towards defibrillation. Those who were satisfied with defibrillation training during their undergraduate studies were more confident as members and leaders of the team,* Nontechnical Skills* scale [*F*(2, 123) = 3.94, <0.01]. Adequate education about recognising the rhythms had even greater effect on their self-confidence scale of* Nontechnical Skills* [*F*(2, 123) = 8.98, <0.0001]. Respondents who had participated in CPR-D training less than 6 months ago scored lower on* Nurse's Role* scale than those having participated over a year ago ( 5.32 SD1.01 versus 5.58 (SD0.84)) (*p* < 0.05). Age or working experience had no effect on the attitudes (Table 4).

The attitudes of the trainers towards guidelines in general were positive as were their thoughts about their competence to use them but the respondents did not think very highly about the availability or practicality of the guidelines (Scale 2: Restrictions, Scale 3: Personal and Organisation) (Table 2). Of the trainers 19.3% (24/124) agreed somewhat or totally that resuscitation guidelines challenge the autonomy of care providers, 26.6% (33/124) agreed that resuscitation guidelines oversimplify medical practice, 35.4% (44/124) thought that resuscitation guidelines are difficult to find if needed, and 19.3% (24/124) have not seen the guidelines in the unit they have last worked in.

Participants working at hospital wards were more sure of themselves as leaders and members of the group than those working in primary care (scale of* Nontechnical Skills* mean 5.70 SD 0.87 versus 5.27 SD 0.93, *p* < 0.05). Eleven percent of the respondents have manual defibrillators at their work. Those having manual defibrillators graded themselves higher in the* Nontechnical Skills* scale than their peers (scale mean 5.68 SD 0.92 versus 6.35 SD 0.69, *p* < 0.01).

Significant associations were observed between scales of* Hesitation*,* Nurse's Role*, and* Nontechnical Skills* (Table 5). Those who were confident about their skills as members and leaders of the group (*Nurse's Role* scale) found the guidelines more useful than the others (*p* < 0.05). Those who felt that their professional competence was not adequate (*Restrictions* scale) scored higher on scale of* Hesitation* (*p* < 0.05) and lower on scales of* Nurse's Role* (*p* < 0.01) and* Nontechnical Skills* (*p* < 0.01), found the Guidelines less* Useful* (*p* < 0.01), and were more concerned about the* organisational* attitudes (*p* < 0.01) than the others.

## 4. Discussion

The findings of associations concerning attitudes towards defibrillation and Current Care Guidelines for CPR were due to differences in location of work, last participation in training, and undergraduate education. Current cardiopulmonary resuscitation guidelines were highly valued among trainers, which are in accordance with previous studies [3, 4, 7, 21, 22]. Unlike the nursing students and nurses [4–7], most trainers felt competent to follow the procedures recommended in the guidelines. Positive attitudes do not necessarily correlate with practice [14, 22, 23], but the trainers' positive attitudes towards nurse-performed defibrillation might help in implementing the practice guidelines of cardiopulmonary resuscitation [14, 21, 24, 25]. Participants hesitating over defibrillation were more sceptical about the usefulness of the Current Care Guidelines compared to the more self-confident peers. Trainers may transfer their attitudes in classroom [4–7, 11, 14].

Previous studies have shown many reasons for healthcare personnel's reluctance to start CPR-D [2–9, 14, 15]. Interventions in the institutions and hospitals have not been successful in increasing the nurses' confidence [4, 6]. The CPR-D refresher courses and standardised training have not been sufficient to provide nurses with adequate skills [5]. According to the results of previous studies, standardised training sessions have not succeeded in addressing the underlying reasons for the nurses' reluctance. In accordance with the present study the participants who had most recently attended a refresher course were still unsure of nurse's role as first respondent.

The education of trainers may benefit from increased educational focus on the defibrillation, recognition of the rhythms, and training of leadership skills because nurse educators may serve as positive role models to encourage and reinforce the crucial role nurses may play as the first healthcare professionals on the scene [14, 21, 26–28].

*Limitations*. The results reported in this study reflect attitudes of the participants working in hospitals and institutions. It can be argued if the results can be extrapolated to other organisations or institutions determined to improve their education. Usually the problems are similar, but the local circumstances vary. However, the authors believe that other investigators find the instruments described in this study as applicable.

The major limitation in the study is that the response rate reached only 67%. Also the main limitation was related to small size of study groups and thus the credibility of results. An implicit flaw in survey-based research is that there may be differences between subjects who chose to respond and those who did not, leading to selection bias. In the study, the selection of participants was made in the two seminars in the same way. The authors chose this particular study sample from two different courses in purpose to get as many participants as possible. Coordinators often work alone and a seminar like this was seen as an excellent opportunity to meet most of the coordinators working in the area. All CPR trainers who were attending seminars were recruited to the study. Responses may not be representative of all CPR trainers but they represented geographically large part of the country.

## 5. Conclusion

Most of the trainers were unsatisfied with their previous CPR-D education and some feel uncertain of defibrillation. Quality of undergraduate education may affect the work of CPR-D trainers. The train-the-trainers courses should be tailored according to learners' needs, which should be clearly solicited prior to the session. There should be increased focus on more practical scenarios with defibrillators and nontechnical skills and on nurse's role as first respondent.

Further research is needed to evaluate the quality of healthcare professionals CPR-D skills in order to identify shortcomings of basic education and CPR-D training among different groups of professionals. One of the interesting topics for further research would be to determine if the attitude of the trainers affects the trainees attitudes.

## Figures and Tables

**Table 1:** Factor loading of the questionnaire distributed to the participants consisting of 58 items using maximum likelihood and varimax rotation. Eigenvalues, total variance explained by factors, and Cronbach's alphas for scales are presented in the table.

	1	2	3
*Scale 1: Hesitation*			
I hesitate to perform defibrillation, because I am not ready	.907		
I hesitate to perform defibrillation, because I do not want to take the lead of the situation	.893		
I hesitate to perform defibrillation, because the resuscitation team is on their way	.883		
I hesitate to perform defibrillation, because the patient might die and I would feel guilty	.849		
I hesitate to perform defibrillation with the device we have available	.842		
I hesitate to perform defibrillation, because I fear to injure the patient	.792		
I am able to perform defibrillation	−.725		.414
I hesitate to perform defibrillation, because I feel the anxiety of the situation	.682		
I feel that a doctor should perform defibrillation	.580		
I hesitate to perform defibrillation, because I am not sure that I recognize the rhythm correctly	.557		
*Scale 2: Nurse's Role*			
I feel that the change in nurse's role is positive		.924	
Nurse's role is changing due to the new resuscitation guidelines		.715	
The personnel should be educated to their new role during their undergraduate education		.430	
All healthcare personnel should be able to perform defibrillation, if needed		.286	
I feel that the first person arriving to the resuscitation scene should perform defibrillation		.177	
*Scale 3: Nontechnical Skills*			
I am competent to lead a resuscitation team	−.446		.714
I am competent to work in a resuscitation team	−.421		.794
*Eigenvalues*	7.680	2.109	1.295
*Variance explained (%)*	38.303	10.155	9.647
*Cronbach's alpha*	0.918	0.570	0.802

**Table 2:** Factor loading of the questionnaire distributed to the nurses consisting of 58 items using maximum likelihood and varimax rotation. Eigenvalues, total variance explained by factors, and Cronbach's alphas for scales are presented in the table.

	1	2	3	4
*Scale 1. Usefulness*				
(14) Resuscitation guidelines can improve the quality of health care	.809			
(15) Resuscitation guidelines are a convenient source of advice	.808			
(16) Resuscitation guidelines are useful as educational tools	.651			
(17) Resuscitation guidelines are based on scientific evidence	.598			
(18) Resuscitation guidelines can improve the interaction between patients and healthcare personnel	.582			
(14) Resuscitation guidelines can improve the quality of healthcare	.434			
*Scale 2. Restrictions*				
(20) Resuscitation guidelines challenge the autonomy of care providers		.830		
(19) To implement resuscitation guidelines is too expensive for us		.703		
(23) Resuscitation guidelines oversimplify medical practice		.534		
*Scale 3. Personal and Organisation*				
(25) Resuscitation guidelines are difficult to find if needed			.974	
(12) My occupational competence is insufficient for adopting the latest resuscitation guidelines			.322	
(21) Resuscitation guidelines are not valued in our organization				.867
(22) Most of our team members have disapproving attitudes about resuscitation guidelines				.477
(24) I have not seen the guidelines in the unit I have last worked in				.440
*Eigenvalues*	4.80	1.90	1.20	1.14
*Variance explained (%)*	34.07	13.53	8.57	8.13
*Cronbach's alpha*	0.804	0.775	0.518	0.648

**Table 3:** Background data of the respondents. A questionnaire was distributed to CPR trainers attending two seminars. Figures are presented as numbers or as percentage.

	Participants (*n* = 124)
Gender (male/female)	17/107
Mean age, mean (SD)	44.2 (9.8)
Working experience in years, mean (SD)	16.9 (9.0)
Physicians	2 (1.6%)
Specialized nurses	33 (26.6%)
Nurses	64 (51.6%)
Paramedics	6 (4.8%)
Other healthcare workers	19 (15.3%)
Working on the ward	90 (80.6%)
Working on the outpatient clinic	24 (19.3%)
Working as CPR coordinator	36 (29.0%)

**Table 4:** CPR training of the respondents. A questionnaire was distributed to CPR trainers attending two seminars. Figures are presented as numbers or as percentage.

	Participants *n* = 124
Gender (male/female)	17/124
Undergraduate CPR training	
Adequate	35 (28.2%)
Not adequate	74 (59.6%)
None at all	5 (4.0%)
No opinion	10 (8.0%)
Undergraduate defibrillation	
Adequate	37 (29.8%)
Not adequate	39 (31.4%)
None at all	48 (38.7%)
No opinion	—
Education about recognising the rhythms	
Adequate	37 (29.8%)
Not adequate	62 (50.0%)
None at all	25 (20.1%)
No opinion	—
Participated in resuscitation training	
<6 months ago	90 (72.5%)
<1 year ago	20 (16.1%)
>1 year ago	14 (11.2%)
No opinion	—

**Table 5:** Correlations between the seven scales of *Hesitation, Nurse's Role, Nontechnical Skill, Usefulness, Restrictions, Personal, and Organisation* (1–3 attitudes towards CPR-D, 4–7 towards AGS). The participants answered using Likert scale 1–7 (1 = totally disagree, 4 = neither agree nor disagree, and 7 = totally agree). Figures are given as scales mean (SD).

	2	3	4	5	6	7
(1) *Hesitation*	−.092	−.513^*∗∗*^	−.262^*∗∗*^	.160	.218^*∗*^	.053
(2) *Nurse's Role*		.142	.126	−.181	−.228^*∗*^	−.026
(3) *Nontechnical Skills*			.283^*∗∗*^	−.198^*∗*^	−.339^*∗∗*^	−.040
(4) *Usefulness*				−.408^*∗∗*^	−.278^*∗∗*^	−.224^*∗*^
(5) *Restrictions*					.370^*∗∗*^	.395^*∗∗*^
(6) *Personal*						.358^*∗∗*^
(7) *Organisation*						—

^*∗*^ *p* < 0.05; ^*∗∗*^ *p* < 0.01.
